# The Changes of Nutrition Labeling of Packaged Food in Hangzhou in China during 2008∼2010

**DOI:** 10.1371/journal.pone.0028443

**Published:** 2011-12-14

**Authors:** Shengfeng Wang, Yong Chen, Miao Liu, Zhiheng Hong, Dianjianyi Sun, Yukun Du, Meng Su, Canqing Yu, Qingmin Liu, Yanjun Ren, Jun Lv, Liming Li

**Affiliations:** 1 Department of Epidemiology and Biostatistics, School of Public Health, Peking University, Beijing, China; 2 Chinese PLA Institute for Disease Control and Prevention, Academy of Military Medical Sciences, Beijing, China; 3 Institute of Geriatrics, Chinese PLA General Hospital, Beijing, China; 4 Division for Chronic and Non-communicable Disease Control and Prevention, Hangzhou Center for Disease Control and Prevention, Hangzhou, China; Fundación para la Prevención y el Control de las Enfermedades Crónicas No Transmisibles en América Latina (FunPRECAL), Argentina

## Abstract

**Objective:**

To understand the changes of the nutrition labeling of packaged food in China two years after the promulgation of the Regulation for Food Nutrition Labeling, which encourages food manufacturers to identify nutrition labeling.

**Methods:**

Investigators copied out the nutrition information panel, nutrition claim and nutrient function claim of packaged food in a supermarket with prepared questionnaire and finished normative judgment in 2008 and 2010.

**Results:**

4693 and 5526 kinds of packaged food were investigated separately. Nutrition information panel, nutrition claim and nutrient function claim were found on the food label of 27.6%, 13.0% and 1.9% of packaged food respectively in 2008, while 35.1%, 7.7% and 2.3% in 2010. The nutrition information panel which labeled energy, protein, fat, carbohydrate and sodium was 597(43.8%) and 1661(85.9%) in 2008 and 2010, only 134(9.8%) and 985(51.0%) nutrition information panel were totally normalized. Nutrition claim and nutrient function claim focused on vitamin, mineral and dietary fiber. The total qualified proportions for nutrition claim were increased significantly for most of the nutrients, except for cholesterol. There were 6 (6.4%) and 5 (3.9%) nutrient function claims with hinting of therapeutic effects on diseases separately.

**Conclusion:**

Although the voluntary regulation remarkably improved the level of normalization for nutrition labeling, its role on the prevalence was minus. It's imperative to enforce nutrition labeling for not only China but also other countries, and furthermore, health education on nutrition labeling should be initiated to support the policy.

## Introduction

In recent years, with the development of the economy and the quickening of life rhythm, consumers all over the world are increasingly demanding packaged foods, which offer convenience and easy accessibility. The consumption of packaged foods in most countries increased year by year, especially in China, of which retail sales in the packaged food industry reached US$111 billion (RMB 758 billion) in 2008, with a compound annual growth rate of 11.5% [1]. In response to consumers' rapidly changing needs, food industries are developing an increasing variety of processed foods. Many food manufacturers increase flavor by simply adding fats, sugars, and salt to foods [2]. Unfortunately, most consumers are either unaware of the increased health risks associated with some processed foods or lacking the skills to distinguish between healthy and unhealthy foods. Nutrition labels were suggested to be an important means of facilitating choice of and access to nutrient-dense foods by World Health Organization (WHO) in 2003 [3]. To help consumers make healthy food choices, governments should ensure accurate, standardized, and comprehensible information is provided on the content of food items [4].

By the end of 2006, only 10 countries in the world already had mandatory regulations. Nutrition labeling in most of countries like China is voluntary unless the food bear a nutrition claim and/or the food has a special dietary use, while still many had no regulations at all at that moment [5]. Since then, China has spent considerable discussion and exploration on nutrition labeling of foodstuffs. And in 2007, the Ministry of Health of the People's Republic of China promulgated the *Regulation for Food Nutrition Labeling*, which took effect on May 1, 2008 [6]. It was an important step in helping to prevent chronic diseases at a national policy level. The regulation encourages food manufacturers to identify nutrition information panel, nutrition claims, and nutrient function claims on sale product labels. Nutrition information panel must be presented in a tabular format with the heading named “Nutrition Information panel” in the pre-packaged foods directly offered to consumers with a few exemption. A standard function claim cannot be used unless the declared value of the content of a nutrient is in compliance with the claim conditions specified in the regulation. Meanwhile, any nutritional component may be claimed provided that the declared value of a nutritive substance in the food is in compliance with the requirements and restrictive conditions listed in the regulation. Nutrition labeling which are written in Chinese should certainly be truthful, subjective and not be in any deceptive, not to exaggerate the functions. A detailed statement of requirements for nutrition labeling can be found in the regulation [6].

In fact, in at least three countries, cost-benefit analyses have been conducted as part of the process of developing regulation, and have actually been used to support the mandatory approach [5]. However, compulsory nutrition labeling is unlikely implemented in one step refer to the experience of America. Chinese regulation sets a grace period of two years to allow the trade to make necessary adjustments, and will definite the product groups, nutrients and the executive date of compulsory labeling according to its implementation status. It has been two years since the regulation promulgated and the grace period is over. Then, how is the practical effect of carrying out the regulation? A study is needed to evaluate its effect so as to further improve the regulation.

Unlike the United States, which uses Food Label and Package Survey (FLAPS) as tracking mechanism to keep abreast of the market response to regulation via changes in product package labels, China has no related national nutrition monitoring program. But fortunately, a survey was conducted soon after the aforementioned regulation was put into place, as part of the Community Intervention for Health (CIH) that has been described in detail elsewhere [7]. And shortly after the grace period, the survey was redone with the same method. This study compares the data completed before and after introduction of the regulation to address 2 questions: (*1*) Has there been an increase in the proportion of packaged food displayed nutrition labeling? (*2*) Has there been improvements in the normalization of the nutrition labeling? To a certain extent, the results were expected to reflect the most common food products that urban residents could buy.

## Methods

### Food product sampling

The study was conducted in one large supermarket in Hangzhou City, which is the capital of Zhejiang Province and located in the eastern part of China. The population of permanent residents was nearly eight million in 2008, of which 69% lived in urban areas. Hangzhou City's comprehensive economic strength ranked eighth among all large- and medium-sized cities of China in 2008 [8].

The national and Hangzhou local food sales database couldn't be found to serve as the sampling frame of food products. As such, a large supermarket was identified, which is in an urban area and belongs to a major supermarket chain that operates 4,930 retail stores in 22 provinces of China. This large supermarket offers a wide variety of foods that, to a certain degree, include most common and popular food brands and items in the country and region. So the aim of this study is to provide a snapshot of the relative better situation of the nutrition labeling in China, and also indirectly reflect the limited effect of the regulation, referenced to other countries' experiences [5], [9]. Our survey covered all domestically commercially made packaged food products sold in the supermarket during the survey periods. Baby foods and infant formula, health food, teas without any added ingredients, and drinks with an ethanol content of >0.5% were not included in the survey.

### Data collection

A data collection form was developed to define variables related to the information presented on the food label. The information fell into two categories: (*1*) general food label information (i.e., net quantity, the origin, manufacturing date, shelf life); (*2*) nutrition labeling information (i.e., presence or absence of the nutrition information panel/standardized nutrition claim/nutrient function claims, as well as their contents and formats). Nutrition information panel means a normative form with the name of the nutrient, the content of nutrient and % NRV (Nutrient reference value) of a food, which can be of any size and should be in vertical align with the package baseline, and must include the information on calories and four core nutrients, namely, protein, carbohydrate, fat, and sodium. Nutrition claim refers to a description, declaration or implication of the nutritional properties of a food, such as declaration of energy value, content claim of protein, etc. Nutrition claim includes nutrient content claim and nutrient comparative claim. (Nutrient content claim means a nutrition claim that describes the energy value or the content level of a nutrient contained in a food. The terminology for nutrient content claim includes “contains”, “high”, “low” or “no”, etc. Nutrient comparative claim means a nutrition claim that compares the energy value or the content level of a nutrient in a food with other known foods of the same type. The terminology for nutrient comparative claims includes “add”, “reduce”, etc.) Nutrient function claim means a nutrition claim that describes the physiological role of a nutrient in growth, development and normal function of the body. All surveyed foods were assigned to one of 18 main product groups, which was defined based on the Hygienic Standards for Uses of Food Additives [10], and China Food Composition [11]. All investigators were trained strictly. The questionnaires finished by each investigator were randomly selected with a proportion of 10% to conduct quality control by the trainer.

### Statistical analyses

Packaged foods of following types are exemption from the regulation [6], [12]: (*1*) the food with a statement on the label that the daily intake amount shall be no more than 10 g or 10 ml. Those include: *a*) part of the condiments; *b*) food adjunct and leavening agents; *c*) solid drink without any added ingredients; *d*) gum-based candy; (*2*) the food is fresh or raw in nature, which is packed in a container that contains no other ingredient or without any addition of ingredient; (*3*) the food with total surface area of ≤100 cm^2^; (*4*) non-pre-packed food sold on the site which is usually bought for immediate consumption; and (*5*) bottled drinking water; (*6*) the food with manufacturing date before May 1, 2008 and shelf time being overdue. Considering feasibility issues, the aforementioned items (1), (2), (4), (5) and (6) were kept, and item (3) was simplified for use to exclude those foods with a small label surface area and sold in bulk containers.

Among all eligible food products, firstly, the percentages of products sold bearing nutrition information panel, standardized nutrition claims, and standardized nutrient function claims were compared. Due to the differences in product groups and product brands between two surveys (table 1), standardized rate for each product group was calculated, which was the summation of products of each brand's displaying rate in 2010 and its constituent ratio in the same product group in 2008. Then this standardized rate for each product group was multiplied by its constituent ratio in 2008, and summed together as the standardized rate for total products. Secondly, energy and four core nutrients (protein, fat, carbohydrate, and sodium) are mandatory labeling items on a nutrition information panel, which expression format were also defined in the regulation, e.g., the numeric value should be expressed as an accurate point value with nutrient reference value (NRV), while corresponding standardized unit and rounding interval should also be used. So among the foods with nutrition information panel, not only the percentage of labeling with calories and core nutrients, but also the changes of their normalization in expression format were evaluated. Finally, standardized nutrition claims are outlined in table 2. The changes of their normalization were calculated for each claimed nutrient, as the regulation describes that the value of any nutritional component with nutrition claim should be displayed and in compliance with the claim conditions. Stata® version 10.1 (StataCorp. LD, College Station, Texas, USA) was used to conduct the statistical analysis.

**Table 1:** Basic description of packaged food products in 2008 and 2010.

	2008		2010		Food brands	Products with same foodbrand between 2008&2010	
Product group	Food brands	Food products	Food brands	Food products	same between 2008&2010	2008	2010
	N(percent)	N(percent)	N(percent)	N(percent)	N(percent)	N(percent)	N(percent)
Cereals, tubers, starches	25(3.1)	64(1.3)	39(4.1)	113(2.0)	11(2.3)	46(1.1)	52(1.2)
Legumes	29(3.6)	114(2.3)	35 (3.7)	92(1.7)	16(3.3)	94(2.3)	65(1.5)
Vegetables	49 (6.0)	169(3.4)	62(6.6)	218(3.9)	25(5.2)	126(3.1)	132(3.1)
Mushrooms and seaweed	15(1.8)	38(0.8)	25(2.7)	44(0.8)	8(1.7)	22(0.5)	21(0.5)
Fruit	42(5.2)	303(6.1)	50(5.3)	366(6.6)	26(5.4)	250(6.2)	311(7.3)
Nuts and seeds	48 (5.9)	373(7.5)	33(3.5)	304(5.5)	24(5.0)	300(7.4)	280(6.6)
Meats	61(7.5)	367(7.4)	62(6.6)	485(8.8)	32(6.7)	268(6.6)	367(8.6)
Milk and dairy products	14(1.7)	265(5.3)	19(2.0)	237(4.3)	9(2.0)	236(5.8)	213(5.0)
Eggs	11(1.3)	22(0.4)	16(1.7)	38(0.7)	7(1.5)	18(0.4)	20(0.5)
Seafood	35(4.3)	158(3.2)	37(3.9)	114(2.1)	18(3.8)	91(2.3)	77(1.8)
Baked goods	95(11.7)	606(12.2)	109(11.6)	693(12.5)	55(11.5)	463(11.5)	527(12.4)
Convenience foods	97(11.9)	704(14.2)	104(11.1)	769(13.9)	65(13.5)	611(15.1)	651(15.3)
Snacks	38(4.7)	268(5.4)	55(5.8)	321(5.8)	21(4.4)	226(5.6)	227(5.3)
Beverages	89(10.9)	537(10.8)	108(11.5)	667(12.1)	61(12.7)	473(11.7)	518(12.2)
Frozen beverages	5(0.6)	36(0.7)	14(1.5)	133(2.4)	5(1.0)	36(0.9)	66(1.6)
Cocoa products, chocolate,and candies	58(7.1)	466(9.4)	60(6.4)	403(7.3)	31(6.5)	398(9.8)	307(7.2)
Fats	25(3.1)	144(2.9)	26(2.8)	142(2.6)	14(2.9)	114(2.8)	100(2.4)
Condiments	79(9.7)	329(6.6)	87(9.2)	387(7.0)	52 (10.8)	270(6.7)	317(7.5)
**Total**	815(100.0)	4963(100.0)	941(100.0)	5526(100.0)	480(100.0)	4042(100.0)	4251(100.0)

**Table 2:** Description of normalization for nutrition claim in 2008 and 2010 (N_2008_ = 975,N_2010_ = 666).

Label component	Claim	2008				2010			
		N(%)	Without nutrient value^a^	Number thatthe valuedidn't fulfillthe required conditions^b^^,^ c	Numberthat totally qualified	N(%)	Without nutrient value	Number thatthe valuedidn't fulfillthe required conditions	Numberthat totally qualified
Energy	Energy reduced	0	-	-	-	0	-	-	-
	Low energy	9	5(55.6)	0(0.0)	4(44.4)	3	0(0.0)	1(33.3)	2(66.7)
	No energy	1	0(0.0)	0(0.0)	1(100.0)	0	-	-	-
	Total for energy	10	5(50.0)	0(0.0)	5(50.0)	3	0(0.0)	1(33.3)	2(66.7)
Protein	Low protein	0	-	-	-	0	-	-	-
	Origin of protein, or including protein	59	15(25.4)	21(47.7)	23(39.0)	24	1(4.2)	8(34.8)	15(62.5)
	High or rich in protein	56	22(39.3)	18(52.9)	16(28.6)	45	14(31.1)	11(35.5)	20(44.4)
	Protein enhanced	0	-	-	-	0	-	-	-
	Total for protein	115	37(32.2)	39(50.0)	39(33.9)	69	15(21.7)	19(35.2)	35(50.7)
Fat	No fat or notincluding fat	2	0(0.0)	2(100.0)	0(0.0)	13	0(0.0)	1(7.7)	12(92.3)
	Low fat	39	14(35.9)	6(24.0)	19(48.7)	24	7(29.2)	2(11.8)	15(62.5)
	Fat reduced	12	2(16.7)	0(0.0)	10(83.3)	5	0(0.0)	0(0.0)	5(100.0)
	Lean	0	-	-	-	0	-	-	-
	Skim	2	0(0.0)	0(0.0)	2(100.0)	6	2(33.3)	2(50.0)	2(33.3)
	No saturated fat or not including saturated fat	0	-	-	-	0	-	-	-
	Low saturated fat	0	-	-	-	6	4(66.7)	0(0.0)	2(33.3)
	No or not includingtransfat	8	2(25.0)	0(0.0)	6(75.0)	18	0(0.0)	0(0.0)	18(100.0)
	Total for fat	63	18(28.6)	8(17.8)	37(58.7)	72	13(18.1)	5(8.5)	54(75.0)
Cholesterol	No or not including cholesterol	62	35(56.5)	0(0.0)	27(43.5)	58	35(60.3)	0(0.0)	23(39.7)
	Low cholesterol	2	2(100.0)	-	0(0.0)	1	1(100.0)	0(0.0)	0(0.0)
	Cholesterol reduced	0	-	-	-	0	-	-	-
	Total for cholesterol	64	37(57.8)	0(0.0)	27(42.2)	59	36(61.0)	0(0.0)	23(39.0)
Carbohydrate	Enhanced or reduced	9	2(22.2)	0(0.0)	7(77.8)	0	-	-	-
Sugar	Sugar free or sugar excluded	41	36(87.8)	0(0.0)	5(12.2)	33	23(69.7)	3(30.0)	7(21.2)
	Low sugar	38	34(89.5)	0(0.0)	4(10.5)	25	15(60.0)	1(10.0)	9(36.0)
	Sugar reduced	0	-	-	-	2	1(50.0)	0(0.0)	1(50.0)
	No lactose	3	3(100.0)	0(0.0)	0(0.0)	0	-	-	-
	Low lactose	1	1(100.0)	-	0(0.0)	0	-	-	-
	Lactose reduced	0	-	-	-	0	-	-	-
	Total for sugar	83	74(89.2)	0(0.0)	9(10.8)	60	39(65.0)	4(19.0)	17(28.3)
Dietary fiber	Origin of dietary fiber,or including dietary fiber	53	21(39.6)	11(34.4)	21(39.6)	36	5(13.9)	2(6.5)	29(80.6)
	High or rich in dietaryfiber or good origin	69	30(43.5)	13(33.3)	26(37.7)	53	19(35.8)	14(41.2)	20(37.7)
	Enhanced or reduced	0	-	-	-	0	-	-	-
	Total for dietary fiber	122	51(41.8)	24(33.8)	47(38.5)	89	24(27.0)	16(24.6)	49(55.1)
Sodium	No sodium or notincluding sodium	0	-	-	-	7	0(0.0)	6(85.7)	1(14.3)
	Low sodium	3	3(100.0)	-	0(0.0)	1	1(100.0)	0(0.0)	0(0.0)
	Sodium reduced	0	-	-	-	0	-		
	Total for sodium	3	3(100.0)	-	0(0.0)	8	1(12.5)	6(75.0)	1(12.5)
Vitamin	Origin of vitamin X or including vitamin X	139	79(56.8)	-	-	65	4(6.2)	-	-
	High or rich in vitamin X	83	31(37.3)	-	-	53	8(15.1)	-	-
	Enhanced or reduced vitamin X	32	7(21.9)	-	-	13	2(15.4)	-	-
	Multivitamin	7	1(14.3)	-	-	12	1(8.3)	-	-
	Total for vitamin	261	118(45.2)	-	-	143	15(10.5)	-	-
Mineral	Origin of X or including X	121	24(19.8)	-	-	56	0(0.0)	-	-
	High or rich in X	94	36(38.3)	-	-	76	19(25.0)	-	-
	Enhanced or reduced	30	7(23.3)	-	-	31	4(12.9)	-	-
	Total for mineral	245	67(27.3)	-	-	163	23(14.1)	-	-
Total for all		975	412(42.3)			666	166(24.9)		

Note:

a Because the vitamin and mineral were always claimed broadly, and seldom subdivided into specific vitamin or mineral, we simplified this to calculate the percentage with nutrition information for this two nutrients and might overestimate the value.

b The number of the numeric value for energy, fat, sugar and sodium with “<”, “≤” or range were 2, 5 and 10 in 2008, while which were 2, 4 and 1 in 2010. For those negative factors, we used the maximum value. Meanwhile, the number of the numeric value for protein and dietary fiber with“>”, “≥” or range were 3, 9, and 1 in 2008, while which were 0, 9 and 4 in 2010. The minimum values for those positive factors were used.

c Excluding vitamin and mineral, there were only 21 and 7 nutrient comparative claims in 2008 and 2010, respectively. Because a “normal food” should be chosen to evaluate whether it fulfilled the content demand, those few nutrient comparative claims were deemed qualified to simplify this question.

## Results

The final databases consisted of 4,693 and 5,526 food products, belonging to 815 and 941 food brands in 2008 and 2010, respectively (the same brand in different product groups was regarded as different brands). 480 food brands were same between 2008 and 2010, corresponding to 4,042 (86.1%) and 4,284 (76.9%) food products separately (table 1). A total of 98 food brands (13.4%) containing 1,151 food products (23.2%) were made by local manufacturers in 2008, while it was 98 food brands (13.4%) including 1,553 food products (28.1%) in 2010.

### Nutrition information panel

An estimated 35.1% of regulation-regulated products sold had nutrition information panel, and the standardized rate was 36.2% in 2010. It indicated a relative small increase from 2008 with a percentage of 27.6%. Different product groups had a wide range of displaying percentages (table 3).The 3 product groups with the highest percentage in both surveys were milk and dairy products, beverages, and fats, respectively.

**Table 3:** Comparison of prevalence of packaged food products with nutrition information panel, nutrition claims and nutrient function claims between 2008 and 2010, by product groups (N_2008_ = 4963, N_2010_ = 5526).

Product group	2008			2010			Standardization for 2010^a^		
	Nutrition information panel	Nutritionclaims	Nutrientfunction claims	Nutrition information panel	Nutritionclaims	Nutrient function claims	Nutrition information panel	Nutrition claims	Nutrient function claims
	N(percent)	N(percent)	N(percent)	N(percent)	N(percent)	N(percent)	(%)	(%)	(%)
Cereals, tubers, starches	20(31.3)	2(3.1)	0(0.0)	29(25.7)	2(1.8)	4(3.5)	31.1	2.9	1.8
Legumes	26(22.8)	7(6.1)	0(0.0)	13(14.1)	1(1.1)	0(0.0)	14.9	1.2	0.0
Vegetables	24(14.2)	6(3.6)	0(0.0)	22(10.1)	0(0.0)	2(0.9)	9.8	0.0	0.6
Mushrooms and seaweed	7(18.4)	4(10.5)	0(0.0)	15(34.1)	7(15.9)	0(0.0)	34.1	21.6	0.0
Fruit	27(8.9)	14(4.6)	1(0.3)	48(13.1)	6(1.6)	2(0.5)	14.2	2.1	1.0
Nuts and seeds	13(3.5)	13(3.5)	0(0.0)	9(3.0)	2(0.7)	0(0.0)	2.7	1.0	0.0
Meats	21(5.7)	8(2.2)	0(0.0)	63(13.0)	21(4.3)	2(0.4)	12.8	4.3	0.5
Milk and dairyproducts	248(93.6)	78(29.4)	27(10.2)	232(97.9)	45(19.0)	8(3.4)	97.6	18.6	3.2
Eggs	0(0.0)	2(9.1)	0(0.0)	1(2.6)	2(5.3)	0(0.0)	1.0	5.6	0.0
Seafood	27(17.1)	19(12.0)	0(0.0)	16(14.0)	7(6.1)	0(0.0)	10.4	6.0	0.0
Baked goods	212(35.0)	86(14.2)	19(3.1)	285(41.1)	45(6.5)	22(3.2)	39.9	6.6	3.4
Convenience foods	159(22.6)	116(16.5)	17(2.4)	308(40.1)	105(13.7)	46(6.0)	37.4	12.9	7.0
Snacks	52(19.4)	25(9.3)	6(2.2)	159(49.5)	15(4.7)	19(5.9)	49.4	3.8	6.5
Beverages	281(52.3)	137(25.5)	21(3.9)	429(64.3)	100(15.0)	22(3.3)	64.4	17.0	7.0
Frozen beverages	14(38.9)	0(0.0)	0(0.0)	30(22.6)	0(0.0)	0(0.0)	53.7	0.0	0.0
Cocoa products, chocolate, andcandies	127(27.3)	41(8.8)	0(0.0)	145(36.0)	10(2.5)	0(0.0)	46.8	5.6	0.0
Fats	73(50.7)	64(44.4)	0(0.0)	73(51.4)	50(35.2)	0(0.0)	51.2	36.3	0.0
Condiments	38(11.6)	21(6.4)	3(0.9)	60(15.5)	6(1.6)	1(0.3)	18.9	3.5	0.4
Total	1369(27.6)	643(13.0)	94(1.9)	1937(35.1)	424(7.7)	128(2.3)	36.2	8.5	2.9

Note:

a standardized rate for each product group was the summation of products of each brand's labeling rate in 2010 and its constituent ratio in the same product group in 2008, then multiplied this standardized rate for each product group by its constituent ratio in 2008 and summed the products as the standardized rate for total products.

The sample decreased to 1364 (the number of products sold had nutrition information panel in 2008) and 1933 (the number of products sold had nutrition information panel in 2010) in the next analysis, due to there were 5 and 4 nutrition information panel blocked in 2008 and 2010, respectively. The regulation allows the nutrition labels in either per 100 g, per 100 ml or per serving format, the number of the products with none of the above was 22 (1.6%), 26 (1.3%) in 2008 and 2010 separately. The percentage of displaying calories and core nutrients was 43.8% in 2008, of which only 22.4% were totally qualified in expression. In 2010, it rose to 85.9%, while 59.4% of which were totally qualified (table 4). Moreover, the regulation proposes the following order for reporting the nutritional components: calories, protein, total fat, total carbohydrate, sodium. The proportion that wasn't in accordance with the order requirements was 42.9% after excluding those with only one nutrient, and it reduced to 12.5% in 2010. As to the regulation, energy and core nutrients should be highlighted with appropriate measures, if there are other nutritional components listed in the form. There were 536 (79.5%), and 568(57.7%) nutrition information panel without meeting above requirements in 2008 and 2010, respectively.

**Table 4:** Description of normalization for nutrition information panel in 2008 and 2010 (N_2008_ = 1364, N_2010_ = 1933^a^).

Nutrients	2008						2010					
	Total	With non-standard units	Without NRV	With non-standard form	With non-Standardrounding	Total qualified^b^	Total	With non-standardunits	Without NRV	With non-standard form	With non-standard rounding	Total qualified
	N(percent)	N(percent)	N(percent)	N(percent)	N(percent)	N(percent)	N(percent)	N(percent)	N(percent)	N(percent)	N(percent)	N(percent)
Energy	1112(81.5)	109(9.8)	667(60.0)	49(4.4)	68(6.1)	442(39.7)	1860(96.2)	81(4.4)	369(19.8)	94(5.1)	55(3.0)	1471(76.1)
Protein	1264(92.7)	44(3.5)	811(64.2)	194(15.3)	575(45.5)	250(19.8)	1869(96.7)	33(1.8)	373(20.0)	127(6.8)	462(24.7)	1198(62.0)
Fats	1264(92.7)	41(3.2)	726(57.4)	181(14.3)	604(47.8)	261(20.6)	1875(97.0)	21(1.1)	354(18.9)	129(6.9)	565(30.1)	1110(57.4)
Saturated fats	225(16.5)	5(2.2)	105(46.7)	23(10.2)	134(59.6)	39(17.3)	275(14.2)	6(2.2)	113(41.1)	12(4.4)	85(30.9)	133(47.8)
Unsaturated fats	43(3.2)	3(7.0)	43(100.0)	0(0.0)	35(81.4)	0(0.0)	82(4.2)	5(6.1)	81(98.8)	9(11.0)	25(30.5)	0(0.0)
Trans fats	89(6.5)	5(5.6)	74(83.1)	2(2.2)	72(80.9)	0(0.0)	67(3.5)	0(0.0)	55(82.1)	3(4.5)	60(89.6)	0(0.0)
Cholesterol	211(15.5)	48(22.7)	110(52.1)	14(6.6)	18(8.5)	88(41.7)	183(9.5)	29(15.8)	65(35.5)	1(0.5)	13(7.1)	102(54.8)
Carbohydrate	1091(80.0)	30(2.7)	552(50.6)	58(5.3)	591(54.2)	267(24.5)	1851(95.8)	25(1.4)	332(17.9)	86(4.6)	328(17.7)	1339(69.3)
Sugar	163(12.0)	2(1.2)	160(98.2)	3(1.8)	115(70.6)	0(0.0)	170(8.8)	6(3.0)	165(97.1)	9(5.3)	52(30.6)	0(0.0)
Fiber	299(21.9)	17(5.7)	138(46.2)	22(7.4)	155(51.8)	63(21.1)	333(17.2)	10(3.0)	99(29.7)	29(8.7)	86(25.9)	182(54.2)
Sodium	637(46.7)	28(4.4)	152(23.9)	10(1.6)	58(9.1)	462(72.5)	1695(87.7)	36(2.1)	182(10.7)	21(1.2)	113(6.7)	1454(75.2)
Calcium	473(34.7)	99(20.9)	318(67.2)	68(14.4)	56(11.8)	152(32.1)	529(27.4)	31(5.9)	153(28.9)	55(10.4)	35(6.6)	364(68.8)
Vitamin A	253(18.5)	190(75.1)	188(74.3)	53(20.9)	30(11.9)	48(19.0)	174(9.0)	78(44.8)	50(28.7)	16(9.2)	9(5.2)	85(48.9)

Note:

a there were 5 and 4 nutrition facts label blocked in 2008 and 2010, respectively, so the sample decreased to 1364 and 1933.

b the regulation defined the expression format for the nutrition component (e.g., expression units, nutrient reference value(NRV), numerical form and rounding interval), “total qualified ” means fulfill all above four requirements.

### Nutrition claims

Among all eligible food products, 643 (13.0%) carried 975 standardized nutrition claims in 2008. In 2010, there were 424 (7.7%) products with 666 claims, and the standardized rate was 8.5%. In both 2008 and 2010, the percentage was highest in fats, milk and dairy products, and beverages(table 3), and among the total nutrition claims, the top three displaying proportions were vitamin, mineral, and dietary fiber. Most of the claim modes were “origin of X or including X”, and “high or rich in X or good origin”. The percentage of the claimed nutrient without declared value was 42.3% in 2008, and it decreased to 24.9% in 2010. The total qualified proportions were increased significantly for most of the nutrients, except for cholesterol (table 2).

### Nutrient function claims

Only 94 (1.9%) products displayed 288 standardized nutrient function claims in 2008, while there were 128 (2.3%) products with 412 claims in 2010, and the standardized rate was 2.9%. In both 2008 and 2010, the percentage was highest in fats, milk and dairy products, and beverages (table 3), and among the total claims, the top three displaying proportions were vitamin, mineral, and dietary fiber. All other nutrition component accounted for merely 3.4% and 7.2%, respectively. There were 6 (6.4%) and 5 (3.9%) nutrient function claims with hinting of therapeutic effects on diseases separately.

## Discussion

This study focuses on the change of the prevalence and normalization for the nutrition labeling of packaged foods in the urban area subsequent to the regulation of China. It is apparent from the unsatisfactory status that a significant amount of the food industries still don't provide nutrition information panel as specified in the regulation. In addition, finding of two surveys indicates that close to 10% of the packaged products have standardized nutrition claims, and only 2% of the products display nutrient function claims.

Typical objectives of the Chinese regulations have been to provide consumers with information, to help consumers make healthful choices, and to encourage food manufactures to develop healthy food products [6]. At the policy formulation stage, Chinese government set a two-year grace period to allow the trade to make necessary adjustments. This study indicated that the prevalence of nutrition information panel and nutrient function claims appeared to have increased, while the proportion of packaged food with nutrition claims slightly reduced after two-year grace period. It should be said that the regulation took effect to a certain extent in improving the percentages of foods having nutrition labeling. Nonetheless, the management works of nutrition labeling still have very long way to want in China, compared with some developed countries, such as Australia, New Zealand and United States, of which the prevalence of nutrition information panel, nutrition claims and nutrient function claims has already reached 100%, 40% and 6%, respectively [9], [13]–[15]. Three points should be emphasized to further improve the regulation in China. Firstly, the effective promotion of the regulation need to develop relevant supporting measures in time, for instance, propaganda work and training. Only with these, numerous food manufacturing companies will be promoted to be familiar with the regulation as soon as possible. Secondly, it looks that some food industries in China have already known nutrition labeling, however, they haven't recognized the importance of displaying it [16]. Except for that a portion of the food products sold in China might not fulfill the claim requirements, some other industries whose food do meet the claim conditions, also didn't display the label claims. For example, 41 beverages, as 75% of the total soft drinks meeting the “low sugar” claim, failed to display this claim in our study. Thirdly, the whole effect of the regulation is undesirability, which is probably relative to its voluntary nature. Take the United States as an example, the Nutrition Labeling and Education Act (NLEA) that was enacted by FDA in U.S. in 1990 was also to encourage enterprises to display the nutrition label, and the prevalence merely increased from 65.9% to 75.5% during the following four years. However, after FDA took the nutrition labeling of most food as mandatory in 1994, the prevalence increased to 96.5% rapidly in 3 years (the additional 3.4% were not in the mandatory list), which reflected that the legal force of the mandatory regulation is larger than that of an encouraging one [14]. In fact, although nutrition labeling was recommended be voluntary unless a nutrition claim is made by the Codex Alimentarius Commission, as an effective means of helping consumers to make health food choices, it was mandatory to display in many developed countries, which could not only increase the nutrition information at the decision point, but also encourage food manufacturers to develop more healthful food products with lower quantities of less healthy nutrients [5], [17]. The mandatory nutrition labeling is certainly impossible to accomplish in one move, and it is worth pleased to see that Chinese regulation plans to take mandatory method at the policy formulation stage, and will definite the product groups, nutrients and the executive date of compulsory labeling according to its implementation status [6]. Notably, a delay of one year to the introduction of mandatory nutrition labeling will have significant adverse impacts on health [18], so its implementation should be the faster the better.

The role of the regulation on the prevalence of nutrition labeling was relative small; however, it remarkably improves normalization. As for energy and four core nutrients that are mandatory labeling items on a nutrition information panel, not only the content that the nutrition information panel displaying was more integrated, but also the number of the form complying with the requirements was much more. It seemed that the regulation provides a reference for the industries willing to display the label, whereas, it has few legally binding on the industries still unwilling to do this work. Moreover, compared with the dramatic improvement of the mandatory labeling items, other voluntary labeling items, such as sugar and trans fat, were still with lower prevalence and worse normalization, which highlighted the limitation of a voluntary policy that couldn't arouse industry's enough attention again. In fact, the normalization of the current nutrition labeling in China was obviously not quite as good, and still exist many problems as our results indicated. The difficulty of effort in using food label information is magnified if the label itself is not well designed, so the information that is presented must be given in the clearest and simplest manner possible [19], and improvements are still needed in China.

Although a variety of approaches was taken in the nutrition claims regulation in different countries [5], it is indisputable that consumers can more easily identify foods that are particularly suitable to them by considering label claims. For example, foods labeled as “low sodium” or “no sodium” will help consumers with restricted sodium diets identify appropriate foods. Many food manufacturer in Europe and America countries always take advantage of label claims to propagandize the virtue of their products [5]. Because the consumer's interest in specific nutritional components plays a leading role in featuring the desirable characteristics of products for the manufacturers [20]–[22], claims partly reflect most of the public's nutrient concerns as well as the Chinese population's diet-related health perspectives. Our data indicated that most of the claims have centered on vitamin, mineral and dietary fiber, which accounted for 60% of the nutrition claims, and 90% nutrition health claims. Neither the public nor the food manufacturers in China are paying enough attention to calories, fats, sugars, and sodium in packaged food. Take the trans fat that cause great concern recently as an example [23], amount statements about trans fat were found on the food label of 12% of the products in the USA in 2006. And only less than 1% of the products were with tans fat claim from our data. However, these negative factors are actually the nutrients that contribute to the development of chronic diseases [19]. It seemed important to Chinese to note the distinction between the values of a nutrient itself and that of information about that nutrient. Therefore, comprehensive nutrition education campaigns should be developed in order to help consumers to form correct health concept, which could not only arouse the people's initiative to label the claim, but also guide the industries' displaying trends of the claim, and this virtuous cycle could promote the claim management. Exactly, many countries recognized the importance of promoting to the public the benefits to be derived from food labels and educating them on how to read the nutrition information on the labels. And in Hong Kong, a special Task Force on Nutrition Labeling Education has also been set up to coordinate public education and promotion activities on nutrition labeling in 2008 [24]. But very few similar works was in Chinese mainland and it's really desperately needed in order to improve the popularity of the nutrition labeling.

This study hopes to provide some advice for Chinese government to improve the management of the nutrition labeling, but the greatest meaning is to reveal to other countries which develop nutrition labeling just at the beginning, that only a compulsory national nutrition policy could ensure the availability of adequate supplies of safe and nutritious foods as well as provide consumers with the educational means for making informed food choices, and related education campaigns is also needed to ensure the effectiveness of the policy. Furthermore, based on American experience, a comprehensive national food label survey should be conducted to provide the baseline information before the regulation, and it is probably best to repeat survey regularly to provide accurate, timely feedback to the government [25].

This study had several limitations. First, nutrition claim is not only with flexible location, but also in variety of forms, which increase the difficulty in its judgment, and may have led to an underestimate. However, it is comparable between two surveys because of the same method. Secondly, the size of nutrition labeling, which are crucial to define if the label helped consumers make informed choices, weren't described in this study, due to that the regulation in China doesn't define these at all. Thirdly, FLAPS sampled food products considering annual sales dollars and used the sales data to weigh FLAPS data [13]–[14], [25]. As the sales data of packaged food products are unavailable in China, equal weight was given to each food product. Lastly, any generalization of food products in the marketplace is limited by the fact that the survey was only conducted in a supermarket. It is concluded from other countries' experiences that food products sold in small stores or stores in rural areas were faced with a worse situation of nutrition labeling, because they sell increasing amounts of less well-known food brands, which are produced by small-scale local food manufacturers [9]. The results of this study maybe only reflect the most common food products that urban residents could buy, and indirectly indicate the situation of nutrition labeling from the relative better aspect. Nevertheless, the survey is the first to provide a snapshot of the change of nutrition labeling among common packaged food products sold in Hangzhou during the grace period of the regulation.

In conclusion, the survey provides the Chinese government with a view of the status of food labels 2 years after implementation of the nutrition labeling regulation. Our study tells many countries that developing nutrition labeling regulation at the initial stage, that, the enforcement is much more effective, and the soon the better, meanwhile, the health education towards industry and customers should be followed in time. Only this twin approach—compulsory regulation plus health education—would make the food manufactures put real emphasis on the nutrition labeling, encourage consumers using this in self-quantifying their intake, and achieve objectives of the regulation.
